# Feline Cutaneous Mycobacteriosis Caused by *Mycobacterium farcinogenes*: The First Case Report from Asia

**DOI:** 10.3390/ani16172734

**Published:** 2026-09-02

**Authors:** Thapanee Chuenngam, Warangkana Promsatit, Chaichan Khamduang, Kotchaphan Vilaiwan, Kochaporn Kanjanasri

**Affiliations:** 1 Dermatology Center, Kasetsart University Veterinary Teaching Hospital, Faculty of Veterinary Medicine, Kasetsart University, Bangkok 10900, Thailand; 2 Intensive Care Unit, Kasetsart University Veterinary Teaching Hospital, Faculty of Veterinary Medicine, Kasetsart University, Bangkok 10900, Thailand; 3 Outpatient Department, Kasetsart University Veterinary Teaching Hospital, Faculty of Veterinary Medicine, Kasetsart University, Bangkok 10900, Thailand

**Keywords:** cats, MALDI-TOF MS, *Mycobacterium farcinogenes*, non-tuberculous mycobacteriosis

## Abstract

A 4-year-old spayed female domestic shorthair cat was referred with history of 8 months of chronic, non-healing draining wounds on the left lateral thigh and left inguinal region. The initial lesions arose from a cat bite wound on the thigh and a concurrent inguinal subcutaneous mass, both of which had failed to respond to empirical antimicrobial therapy. Prior to referral, cytology of the inguinal mass suggested a spindle cell tumor; however, histopathology following surgical excision revealed severe necrotizing pyogranulomatous mastitis and dermatitis, with no evidence of neoplasia. At the initial referral visit, bacterial cultures from both lesions yielded *Mycobacterium farcinogenes*, identified via matrix-assisted laser desorption/ionization time-of-flight mass spectrometry (MALDI-TOF MS). The cat was treated with pradofloxacin, topical amikacin gel, and azithromycin despite in vitro resistance, resulting in complete clinical remission after 24 weeks without adverse effects. To our knowledge, this is the first reported case of feline cutaneous mycobacteriosis caused by *M. farcinogenes* in Asia. This case expands the limited veterinary literature on *M. farcinogenes* infection, highlights the diagnostic challenges of atypical mycobacterial infections, emphasizes the critical role of species-level identification, and demonstrates the efficacy of prolonged, multidrug therapy in managing chronic non-healing wounds in cats.

## Simple Summary

*Mycobacterium farcinogenes* is a rapid-growing non-tuberculous mycobacterium (NTM) that was first recognized as the causative agent of bovine farcy, a chronic suppurative granulomatous disease affecting the skin and lymphatic vessels of zebu cattle in East and Central Africa. In cats, *M. farcinogenes* is an exceptionally rare cause of cutaneous infection. Affected cats typically develop chronic non-healing wounds, subcutaneous nodules, and multiple draining tracts, most commonly involving the ventral abdomen and inguinal region, which occur through contamination of skin wounds, bite injuries, penetrating foreign bodies, or surgical sites. Because the clinical signs resemble many other chronic skin diseases and mycobacteria are often resistant to commonly used antibiotics, diagnosis and treatment can be challenging.

## 1. Introduction

Feline cutaneous mycobacteriosis is an uncommon infectious disease caused by several species of Mycobacterium, including members of the *Mycobacterium tuberculosis* complex (MTBC), fastidious or lepromatous mycobacteria, and non-tuberculous mycobacteria (NTM) [1,2,3,4,5,6,7,8]. These organisms are Gram-positive, aerobic, acid-fast bacilli [1,2,5,9] characterized by a cell wall rich in lipids and mycolic acids, which contributes to their resistance to heat, disinfectants, and decolorization by acid–alcohol during acid-fast staining procedures [5,9].

*Mycobacterium farcinogenes* is a rapid-growing species belonging to the NTM group [10,11,12]. It was first described in 1973 as the causative agent of bovine farcy, a chronic suppurative granulomatous disease affecting the skin and lymphatic vessels of zebu cattle in East and Central Africa [10,11,13,14,15,16]. Infection generally occurs through cutaneous inoculation following skin trauma, wound contamination, or surgical procedures; soil, water, and decaying vegetation are the primary environmental sources of these bacteria [1,2,3,5,6,7,8,9,17,18]. Clinical lesions are typically characterized by subcutaneous nodules, non-healing wounds, and multiple draining tracts, distributed on the ventral abdomen and inguinal regions [1,2,5,7,8,9,17,18,19,20], where the organism replicates within lipid-rich tissues [1,2,3,8,9,18]. In some cases, lesions may also extend to the pelvic limbs and tail base [5,8].

Treatment usually requires prolonged administration of dual or triple antimicrobial agents [3,4,21], newer-generation fluoroquinolones such as pradofloxacin or moxifloxacin, macrolides such as clarithromycin or azithromycin, and doxycycline may also be considered treatment options for some rapid-growing mycobacterial species [1,2,5]. Combination antimicrobial therapy is generally recommended to reduce the risk of antimicrobial resistance [1,2,4,22,23]. However, in vitro susceptibility does not always correlate with clinical response [5,24]. The treatment should be continued for at least one to two months beyond complete lesion resolution [1,2,3,4,5,9]. Surgical excision may be beneficial in selected cases but can be complicated by wound dehiscence and delayed healing [1,2,5,8,9].

Reports of *M. farcinogenes* infection in both humans and animals remain rare, sporadic human cases were described in the literature [11,12,13]. To date, only one feline case of *Mycobacterium farcinogenes* infection has been described in an online educational case report [25]. The present case report describes cutaneous mycobacteriosis caused by *M. farcinogenes* in a cat and documents successful treatment despite in vitro resistance to one of the administered antimicrobial agents. To the authors’ knowledge, this is the first reported case of feline cutaneous mycobacteriosis caused by *M. farcinogenes* in Asia.

## 2. Case Description

### 2.1. History

A 4-year-old spayed female domestic shorthair cat was referred to the Kasetsart University Veterinary Teaching Hospital for evaluation of an 8-month history of chronic non-healing draining wounds involving the left lateral thigh and left inguinal region. The lesion on the left lateral thigh developed following a cat bite wound and failed to respond to routine wound management, empirical antimicrobial therapy, and repeated wound lavage. Aerobic bacterial culture performed by the referring veterinarian yielded no bacterial growth.

Three months after the initial bite injury, tissue swelling was identified in the left inguinal region and adjacent to the left fifth mammary gland. Fine-needle aspiration cytology was suggestive of a spindle cell tumor with inflammation, prompting surgical excision and histopathological examination. At the time of surgery, the thigh wound was debrided and a surgical drain was placed. Following suture removal, the mammary surgical incision developed wound dehiscence with mucopurulent discharge, while the thigh wound remained persistently draining despite treatment with amoxicillin-clavulanic acid (16 mg/kg PO q12h), prednisolone (0.5 mg/kg PO q24h), and routine wound care. Histopathological examination of the excised tissue revealed severe necrotizing pyogranulomatous mastitis and dermatitis. Due to the progressive, non-healing nature of both lesions, the cat was referred for further diagnostic investigation and management.

### 2.2. Physical Examination

At the initial presentation, the cat was bright, alert, and responsive. Vital parameters were within normal limits, and the body condition score was 5/9. Physical examination revealed a chronic non-healing wound at the previous surgical site in the left inguinal region, characterized by a subcutaneous swelling, multiple draining tracts, and mucopurulent discharge. Two additional draining wounds were also present on the left lateral thigh.

### 2.3. Diagnostic Methods

Fine-needle aspiration of the inguinal lesion was performed for cytological examination using Wright–Giemsa staining. Exudate from the draining tracts at both sites was collected for bacterial culture and antimicrobial susceptibility testing. Samples were inoculated onto sheep blood agar, MacConkey agar, Columbia blood agar, and chocolate agar and incubated at 37 °C in 5% CO_2_; colonies were first observed after 2 days of incubation. Visible colonies were subjected to acid-fast staining and, following purification by subculture, to species identification by matrix-assisted laser desorption/ionization time-of-flight mass spectrometry (MALDI-TOF MS; Bruker Biotyper, Bruker Daltonics, Bremen, Germany). A pure subculture of the isolate was used for antimicrobial susceptibility testing by a laboratory-adapted disk diffusion method [26]. Briefly, a bacterial suspension adjusted to a 0.5 McFarland standard was spread onto Mueller–Hinton agar supplemented with 5% sheep blood, antimicrobial disks were applied, and plates were incubated at 37 °C in 5% CO_2_ for 48 h before measuring zone diameters [27]. Because validated species-specific breakpoints for *M. farcinogenes* are not available [27], zone diameters were interpreted using CLSI veterinary interpretive criteria [28] where available, and human CLSI [29] or EUCAST [30] criteria as surrogate standards for agents without veterinary-specific breakpoints. Pending laboratory results, amoxicillin-clavulanic acid at 19 mg/kg PO q12h (Amoxclamed^®^, BIC Chemical Co., Ltd., Bangkok, Thailand) was prescribed.

### 2.4. Laboratory Results

**Figure 1:**
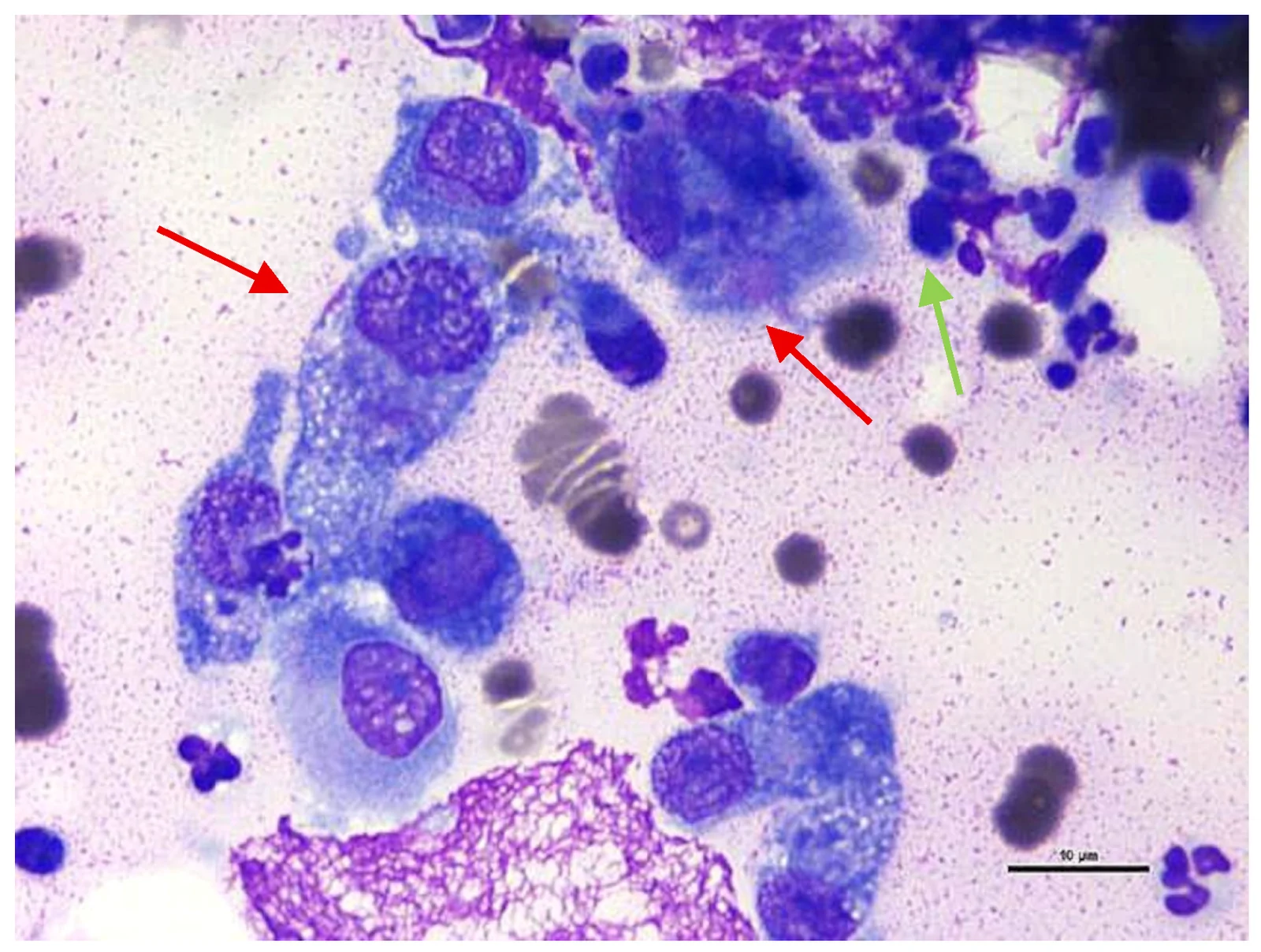
Cytology from fine-needle aspiration of granuloma revealed numerous macrophages (red arrow) admixed with neutrophils (green arrow), consistent with pyogranulomatous inflammation. Wright–Giemsa staining. Magnification bars = 10 µm.

Cytological examination revealed numerous neutrophils and macrophages, consistent with pyogranulomatous inflammation (Figure 1). Bacterial culture of samples collected from both lesions yielded growth of *Mycobacterium farcinogenes*, a rapid-growing non-tuberculous mycobacterium (NTM) that has been rarely reported in cats. Colonies were first visible after 2 days of incubation, and species identification was reported 5 days after sample submission. Acid-fast staining of the isolate demonstrated numerous acid-fast-positive bacilli, and the organism was identified as *M. farcinogenes* by MALDI-TOF MS.

### 2.5. Antimicrobial Susceptibility Testing

Antimicrobial susceptibility testing from both sites demonstrated susceptibility to amikacin, gentamicin, imipenem, meropenem, marbofloxacin, and pradofloxacin, with intermediate susceptibility to amoxicillin-clavulanic acid and enrofloxacin, and resistance to the remaining antimicrobial agents tested (Table 1). Because validated zone-diameter breakpoints for *M. farcinogenes* have not been established, these interpretive categories should be regarded as preliminary phenotypic guidance rather than standardized susceptibility classifications [27].

### 2.6. Treatment

Based on the antimicrobial susceptibility results, pradofloxacin at 6.6 mg/kg PO q24h (Veraflox^®^, Elanco (Thailand) Ltd., Bangkok, Thailand) and topical amikacin sulfate 5% gel (Likacin^®^ gel 5%, S Charoen Bhaesaj Trading, Bangkok, Thailand) applied once daily into the lesions were prescribed. Azithromycin at 8.2 mg/kg PO q24h (Azith^®^, Siam Bheasach Co., Ltd., Bangkok, Thailand) was additionally included in the treatment protocol despite in vitro resistance.

### 2.7. Treatment Outcomes

**Figure 2:**
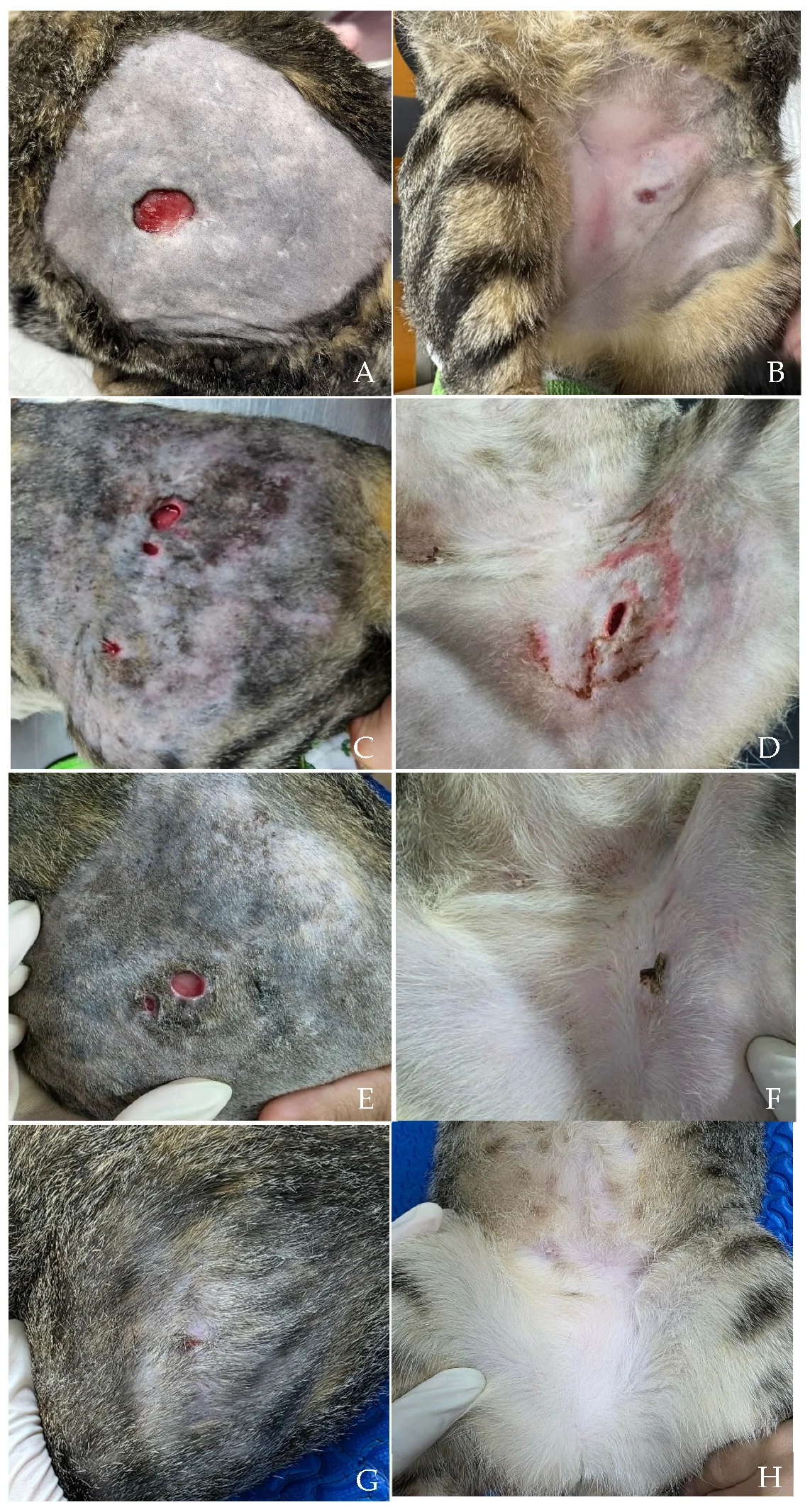

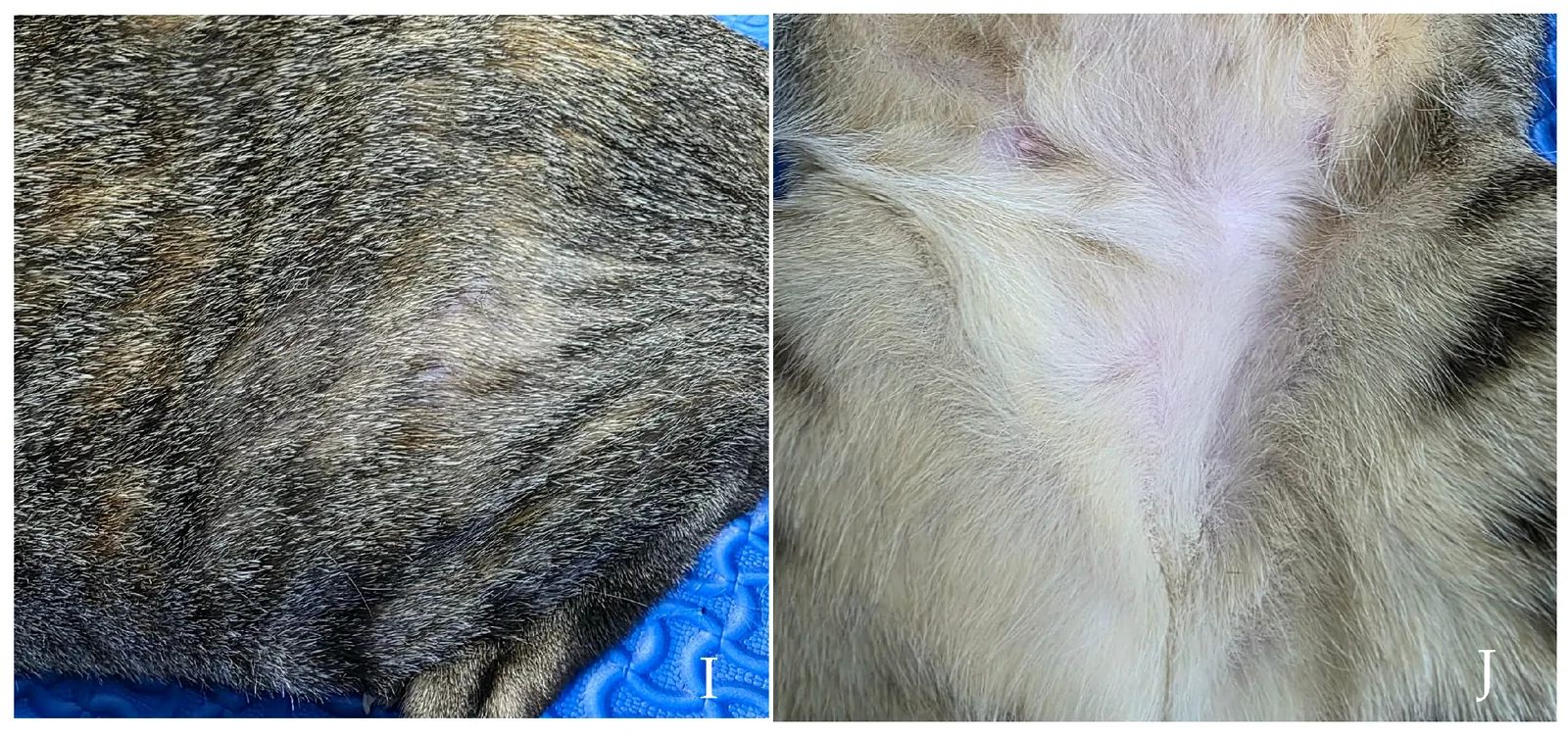
Clinical progression of cutaneous lesions before and during treatment. The left lateral thigh lesion had been present for 5 months before treatment (**A**), followed by the development of an inflamed left inguinal lesion 3 months later (**B**). Clinical photographs were obtained at Day 5 (**C**,**D**), Day 28 (**E**,**F**), Day 119 (**G**,**H**), and Day 175 (**I**,**J**) after treatment initiation.

The lesions gradually improved over time, amikacin gel was discontinued on day 21 after inguinal open wound was healed, and complete clinical remission was achieved after 24 weeks of treatment (Figure 2). Hematological and serum biochemical analyses performed during follow-up remained within normal limits. No adverse effects associated with treatment were observed, and the cat remained clinically well throughout the treatment period.

## 3. Discussion

Feline cutaneous mycobacteriosis is an uncommon infectious disease caused by a diverse group of mycobacterial species, including rapid-growing non-tuberculous mycobacteria (NTM). Although sporadic feline infections caused by rapid-growing NTM—such as *Mycobacterium abscessus*, *Mycobacterium smegmatis*, and members of the *Mycobacterium fortuitum* complex—have been reported, infection caused by *Mycobacterium farcinogenes* appears to be exceptionally rare. *M. farcinogenes* is an environmental rapid-growing NTM species originally recognized as the causative agent of bovine farcy, a chronic granulomatous disease affecting cattle in tropical regions characterized by purulent lymphangitis and cutaneous lesions [10,11,13,14,15,16]. However, bovine farcy has been primarily reported in sub-Saharan Africa; although historical accounts exist in parts of South and Southeast Asia, no cases have been reported in livestock or water buffaloes in Thailand or neighboring Asian countries [15]. In cattle, serological testing has been investigated as an ancillary diagnostic approach, with whole-cell antigen ELISA demonstrating potential utility for the serodiagnosis and epidemiological screening of bovine farcy caused by *M. farcinogenes* [31]. However, the applicability of this approach to feline NTM infections, particularly *M. farcinogenes*, remains undetermined. Reports of infection in companion animals, particularly cats, remain extremely limited, and information regarding its pathogenicity, clinical behavior, and optimal treatment in cats is scarce. To the authors’ knowledge, this is the first reported case of feline cutaneous mycobacteriosis caused by *M. farcinogenes* in Asia. This case provides additional clinical information on the presentation, diagnosis, and treatment of this rare infection in cats.

The lesion distribution in this case is consistent with feline cutaneous mycobacteriosis, which typically targets lipid-rich subcutaneous tissues [1,2,3,8,9,18], causing panniculitis, nodules, ulceration, and draining tracts. These are commonly found on the ventral abdomen, inguinal region, tail base, and pelvic limbs [1,2,5,7,8,9,18,19,20]. In the present case, the initial lesion developed on the left lateral thigh following a reported cat bite wound, and a second lesion subsequently developed in the left inguinal region. Although the exact route of spread could not be determined, rapidly growing mycobacteria are known to extend along subcutaneous tissue planes, and sinus tracts may not always be clinically apparent. Therefore, extension of a pre-existing infection may have contributed to the postoperative wound dehiscence. The chronic bite wound may also have allowed environmental contamination with the organism. This case demonstrates the importance of including mycobacterial infection as a differential diagnosis for cats with chronic, non-healing wounds. When clinically feasible, presurgical diagnosis and management can optimize antimicrobial therapy and prevent complications such as wound dehiscence or delayed healing.

Diagnosing feline cutaneous mycobacteriosis remains challenging [1,3,4,5,25] because the clinical presentation is often non-specific and may closely resemble other chronic dermatological conditions, including bacterial infections, deep fungal infections, foreign-body reactions, immune-mediated diseases, and neoplasia [2,8,32,33]. In the present case, fine-needle aspiration (FNA) cytology of the mammary mass performed before referral was initially interpreted as suggestive of a spindle cell tumor with concurrent inflammation, prompting surgical excision. Histopathological examination revealed severe necrotizing pyogranulomatous mastitis and dermatitis without evidence of neoplasia. In chronic pyogranulomatous inflammation and panniculitis, reactive fibroblasts and myofibroblasts may proliferate during fibroplasia and appear as spindle-shaped cells on cytology, potentially mimicking a mesenchymal neoplasm [34]. Therefore, cytological findings suggestive of spindle cell neoplasia should be interpreted cautiously, particularly when accompanied by marked inflammation, and should be interpreted together with histopathological and microbiological findings. In addition, pyogranulomatous inflammation should prompt investigation for infectious causes using special stains such as acid-fast staining for mycobacteria and periodic acid–Schiff (PAS) or Gomori methenamine silver (GMS) stains for fungal pathogens. In this case, these stains were not performed on the histopathology specimens because the tissue had already been processed before referral. Acid-fast staining was also not performed on cytological smears because it is not used routinely for skin cytology in our laboratory. This represents a limitation of the present report. Acid-fast staining should be considered in cases of pyogranulomatous inflammation, particularly when mycobacterial infection is suspected.

A definitive diagnosis was established by bacterial culture and species identification. An interesting finding in this case was the difference in bacterial culture results before and after referral. The initial culture performed by the referring veterinarian yielded no growth, whereas repeat culture successfully isolated *Mycobacterium farcinogenes* from both lesions. This finding suggests that a negative bacterial culture does not rule out mycobacterial infection. Factors such as sample quality, sampling depth, bacterial load, transport conditions, and laboratory procedures may affect culture results [1,2,5]. Therefore, repeat bacterial culture performed by a specialized laboratory should be considered when mycobacterial infection is still suspected despite an initial negative result. In the present case, visible growth was detected within five days, consistent with the characteristics of rapidly growing NTM, which typically produce colonies within seven days on routine culture media [5,13]. Early bacterial growth enabled prompt species-level identification by matrix-assisted laser desorption/ionization time-of-flight mass spectrometry (MALDI-TOF MS), supporting its value as a rapid, accurate, and cost-effective diagnostic tool for veterinary mycobacteriology [11,35,36]. Because bacterial culture may occasionally be negative despite clinical suspicion, PCR and target sequencing should also be considered, when available, especially if acid-fast staining is negative or culture results are inconclusive [1,2,4]. Although MALDI-TOF MS provided rapid species-level identification during routine diagnostic workflow, 16S rRNA gene sequencing remains the definitive for confirming uncommon mycobacterial species [4,13,17,23]. In this retrospective report, 16S rRNA sequencing could not be performed as the culture isolate was no longer retained post-treatment. In future cases, additional 16S rRNA gene sequencing should be considered to further confirm species identification.

Treatment of feline cutaneous mycobacteriosis is often prolonged and typically requires combination antimicrobial therapy to improve treatment success and reduce the risk of antimicrobial resistance [1,2,3,4,5]. In the present case, prednisolone had been administered for wound management before the diagnosis of cutaneous mycobacteriosis. Corticosteroid therapy should therefore be avoided in suspected mycobacterial infections, as immunosuppression may impair cell-mediated immunity and hinder effective control of intracellular mycobacteria [1,2,37,38].

In the present case, treatment with pradofloxacin, topical amikacin gel, and azithromycin resulted in complete clinical remission after 24 weeks without adverse effects. Pradofloxacin was selected based on in vitro susceptibility and its established efficacy against rapidly growing non-tuberculous mycobacteria [1,2,5], supported by its favorable tissue penetration in deep pyogranulomatous lesions [39]. Although systemic amikacin, including administration at 10 mg/kg SC once daily, represents an important therapeutic option for mycobacterial infections, topical amikacin gel was selected in this case as an adjunctive treatment for the accessible draining lesions, while avoiding prolonged systemic aminoglycoside exposure and its associated risk of nephrotoxicity. However, topical administration may provide limited penetration into deeper subcutaneous tissues, and systemic amikacin may therefore be considered in cases with extensive or deep tissue involvement, with appropriate monitoring.

Regarding azithromycin, although the isolate demonstrated in vitro resistance, it was initially prescribed as part of an empirical multidrug regimen for the extensive subcutaneous lesions [1,2,3,4,5]. It has immunomodulatory properties that may contribute to clinical improvement through modulation of macrophage function [40]. However, given the reliability of susceptibility testing for rapidly growing mycobacteria, the in vitro resistance makes a major contribution from azithromycin less likely. Nevertheless, discrepancies between in vitro susceptibility and clinical response have been reported in mycobacterial infections [5,24], and a supportive contribution from azithromycin cannot be completely excluded in this case. Pradofloxacin was considered the most likely principal contributor to clinical resolution because the isolate was susceptible and the drug was administered systemically throughout treatment. Topical amikacin may also have contributed locally, although it was discontinued after 21 days. Because multiple antimicrobial agents were used concurrently, the individual contribution of each drug cannot be determined. Synergistic activity between azithromycin and pradofloxacin also cannot be established without formal synergy testing. This case emphasizes the importance of using in vitro susceptibility results to guide definitive antimicrobial selection while interpreting treatment response within the broader clinical context of lesion extent, tissue penetration, antimicrobial combinations, and individual host immune responses [41]. No adverse effects were observed throughout the treatment period; serial hematological and serum biochemical evaluations remained within normal limits. Although complete resolution was maintained without recurrence during the follow-up period, long-term monitoring remains advisable due to the known potential for recurrence in feline NTM infections and the limited data regarding the long-term clinical behavior of *M. farcinogenes* in cats.

Overall, this case emphasizes that mycobacterial infection should be included in the differential diagnosis of cats with chronic non-healing wounds, recurrent wound dehiscence, or multiple draining tracts, particularly when empirical antimicrobial therapy is unsuccessful. Repeated sampling, species identification, and antimicrobial susceptibility testing were valuable for establishing an appropriate treatment plan. As reports of feline *M. farcinogenes* infection remain limited, this case provides additional clinical information that may be useful in similar cases.

## 4. Conclusions

This report documents the first confirmed case of feline cutaneous mycobacteriosis caused by *Mycobacterium farcinogenes* in Asia. The case highlights the importance of considering mycobacterial infection as a differential diagnosis in cats presenting with chronic non-healing wounds and multiple draining tracts, particularly when empirical treatment fails. In this case, complete clinical remission was achieved following prolonged multidrug therapy, although the individual contribution of each antimicrobial could not be determined. Early recognition, species-level identification, and susceptibility-guided treatment may contribute to favorable clinical outcomes in affected cats.

## Figures and Tables

**Table 1:** Antimicrobial susceptibility results determined by disk diffusion.

	Interpretation		
Antimicrobial (Disk Concentration)	S	I	R
Amikacin (30 μg)	✓		
Gentamicin (10 μg)	✓		
Imipenem (10 μg)	✓		
Meropenem (10 μg)	✓		
Marbofloxacin (5 μg)	✓		
Pradofloxacin (5 μg)	✓		
Amoxicillin-clavulanic acid (20/10 μg)		✓	
Enrofloxacin (5 μg)		✓	
Azithromycin (15 μg)			✓
Cefovecin (30 μg)			✓
Ceftriaxone (30 μg)			✓
Cephalexin (30 μg)			✓
Chloramphenicol (30 μg)			✓
Clindamycin (2 μg)			✓
Doxycycline (30 μg)			✓
Florfenicol (30 μg)			✓
Minocycline (30 μg)			✓
Sulfamethoxazole/trimethoprim (23.75/1.25 μg)			✓

S, susceptible; I, intermediate; R, resistant.

## Data Availability

The original contributions presented in this study are included in the article. Further inquiries can be directed to the corresponding author.
